# Knowledge, attitude, and practice of cervical cancer screening among women living with HIV in the Kilimanjaro region, northern Tanzania

**DOI:** 10.1002/cnr2.1374

**Published:** 2021-03-19

**Authors:** Faustini C. Kimondo, Happiness D. Kajoka, Meshack R. Mwantake, Caroline Amour, Innocent B. Mboya

**Affiliations:** ^1^ Community Health Department Institute of Public Health, Kilimanjaro Christian Medical University College Moshi Tanzania; ^2^ Department of Epidemiology and Biostatistics Institute of Public Health, Kilimanjaro Christian Medical University College Moshi Tanzania; ^3^ School of Mathematics, Statistics, and Computer Science, University of KwaZulu‐Natal Pietermaritzburg South Africa

**Keywords:** attitude, cervical cancer, cervical cancer screening, Kilimanjaro, knowledge, women living with HIV

## Abstract

**Background:**

Cervical cancer is the fourth most common cancer globally among women in incidence and mortality. Women living with HIV (WLHIV) are disproportionately at a higher risk of developing the disease.

**Aim:**

To determine the knowledge, attitude, and practice of cervical cancer screening among WLHIV in the Kilimanjaro region, northern Tanzania, following the integration of these services in routine HIV care in the country.

**Methods and results:**

A cross‐sectional study was conducted in the Kilimanjaro region among 297 WLHIV attending care and treatment centers (CTC) in Hai district and Mawenzi regional hospitals in northern Tanzania between 21 August and 3 September 2020. A questionnaire was used for data collection using face‐to‐face interviews. Data were analyzed using SPSS version 20.0. Frequencies and percentages summarized categorical variables and numerical variables summarized using median and interquartile range (IQR). About half (50.2%) of 297 WLHIV in this study had ever screened for cervical cancer, and 64% screened within the past 12 months preceding the survey. Although 90% ever heard of cervical cancer screening, only 20.5% knew when WLHIV should start screening. Over half (52.5%) had adequate knowledge of prevention, 38.4% on risk factors, and 27.9% of cervical cancer signs and symptoms. Two‐thirds (66.7%) had positive attitudes toward cervical cancer screening. A major source of cervical cancer screening information was the health care providers (80.1%) and the mass media (66%), particularly radio.

**Conclusions:**

The WLHIV in this study had inadequate knowledge but favorable attitudes toward cervical cancer screening, while half had screened for cervical cancer. Efforts should be directed to capacity building of health care providers at CTC and scaling up the mass media campaigns as relevant interventions to promote the uptake of cervical cancer screening programs among WLHIV in Tanzania.

## 1 INTRODUCTION

Cervical cancer is a public health concern being the fourth most common cancer among women in incidence and mortality,^1^, ^2^ with 570 000 new cases in 2018 representing 6.6% of all female cancers worldwide.^2^, ^3^ Approximately 90% of all new cervical cancer cases occur in low‐ and middle‐income countries and is also the second most common cancer among women in sub‐Saharan Africa.^1^, ^2^ In 2018, 26 009 women were living with cervical cancer in East Africa, which accounted for 26.2% of all cancer cases among women in the region, Tanzania accounting for 39% of all cases.^1^ The prevalence of cervical cancer among women living with HIV is high and more alarming in sub‐Saharan Africa. Prevalence is estimated to range between 1.3% in Kenya and 6% in Nigeria.^4^, ^5^, ^6^, ^7^, ^8^ Within Tanzania, the prevalence ranges from 7.3% in Mwanza to 11% in Morogoro,^9^, ^10^ higher than that reported in Nigeria. Poor screening practices may partly contribute to the high prevalence of cervical cancer among WLHIV in Tanzania.

Virtually, all cervical cancer cases (99%) are associated with genital infection with high‐risk human papillomavirus (HPV)—a widespread virus transmitted through sexual contact.^2^, ^11^, ^12^, ^13^ WLHIV have a higher risk of developing the disease, mainly due to their immune‐compromised state.^1^, ^9^, ^10^, ^14^, ^15^, ^16^ Knowledge and attitudes toward cervical cancer screening are crucial in determining the screening intervention's uptake among women.^17^, ^18^, ^19^, ^20^ The belief of not being susceptible to cervical cancer, fear of cancer diagnosis, fear of exposing their (women's) private parts, anticipated pain of the testing procedure, a long distance from home to the clinic, poor access to screening results, long waiting time, and fewer healthcare workers are other barriers to cervical cancer screening among women living with HIV.^18^, ^21^, ^22^, ^23^

Despite the heavy burden of the disease, cervical cancer is a highly preventable disease in women, including those living with HIV.^19^, ^24^ WHO introduced comprehensive cervical cancer prevention in 2014, which comprises cervical cancer screening, targeting women who are at higher risk of developing the disease.^24^ With the introduction of mass HPV vaccination for young girls in some developing countries, there are opportunities to offer the vaccine to HIV‐positive middle‐aged women through the existing HIV care and treatment programs.^16^ In Tanzania, cervical cancer screening has been integrated into HIV care and treatment services where screening is initiated soon after HIV diagnosis without regarding the woman's age and is conducted annually.^25^

Despite that, the proportion of HIV‐positive women screened for cervical cancer in CTC is low. In Dar Es Salaam, only 9% of women living with HIV ever had at least one cervical cancer screening test.^26^ However, this study was conducted before the integration of cervical cancer screening in routine HIV care. Since this integration, little has been done to assess the uptake of cervical cancer screening services among women living with HIV in Tanzania. This study aimed to determine the knowledge, attitudes, and cervical cancer screening practices among WLHIV in the Kilimanjaro region, northern Tanzania, following the integration of these services in CTC services. Findings from this study will provide information to assess the efficacy of this program and inform future interventions. These findings may also assist the healthcare providers, particularly at CTC, in promoting the uptake of cervical cancer screening services among WLHIV. Moreover, the study results may contribute to developing policies, guidelines, and strategic decisions that will enhance the current screening practices in this population.

## 2 MATERIALS AND METHODS

### 2.1 Study design, setting, and population

We conducted a health facility‐based cross‐sectional study in the Kilimanjaro region between 21 August and 3 September 2020. The region has 396 health facilities that are 20 hospitals, 41 health centers, and 335 dispensaries. Out of these health facilities, 52 provide care and treatment (CTC) services.^27^, ^28^ CTC is the gateway where people living with HIV can access HIV care, treatment, and support services.^25^ The prevalence of HIV in the Kilimanjaro region was 2.6% and was high (3.1%) among women aged 15 years and above.^29^, ^30^ The study population was all WLHIV in the Kilimanjaro region and were attending CTC at data collection time. The study included all women aged 18 to 55 years who provided informed consent. In Tanzania, WLHIV are supposed to be screened for cervical cancer immediately after HIV diagnosis.^25^ The study excluded severely ill women and had undergone total hysterectomy because severely ill women could not respond to the questions. The women with total hysterectomy had their cervix surgically removed.

### 2.2 Sample size and sampling

The sample size was calculated using the formula for estimating a single proportion, given as (*N* = [*Z*_a/2_]^2^ × *p*[1−*p*]/*e* ^2^), where N is the desired sample size, and p is the estimated prevalence of cervical cancer screening among women living with HIV, assumed to be 20%. Furthermore, e is the margin of error or precision (5%), and *Z* is the standard normal value (1.96) corresponding to a 95% confidence interval. After adding a 10% proportion of nonresponse, the minimum estimated sample size was 271 participants.

A simple random sampling technique was used to select Hai among the rural districts of the Kilimanjaro region. Moshi municipality was purposefully selected to ensure rural–urban representativeness. One CTC in each district with the highest number of women enrolled was selected (Hai district hospital and Mawenzi regional referral hospital). All women who attended the CTCs were selected for inclusion. Sampling was done proportional to the size of each selected CTC.

### 2.3 Data collection methods

Face‐to‐face interviews were used for data collection using an electronic administered questionnaire. The questionnaire was adapted and modified from previous studies.^31^, ^32^ The questionnaire was in both English and Swahili languages. It contained information on participant social‐demographic characteristics, knowledge, and attitudes on cervical cancer screening, cervical cancer screening practices, and HIV care and treatment. Trained doctor of medicine students collected data. The interviews were administered in Swahili (local) language and were conducted in a quiet place around the CTC clinics after obtaining informed consent. Each interview took about 20 to 30 minutes.

### 2.4 Study variables

The primary outcome was cervical cancer screening practice measured by asking women if they had ever screened for cervical cancer or not, the reason for screening, the timing of screening since diagnosed with HIV, and whether they had ever screened in the past 12 months.

Knowledge and attitudes on cervical cancer screening were secondary outcomes. Knowledge of cervical cancer was measured by asking participants if they ever heard cervical cancer and knowledge of causes, signs, risk factors, and prevention. Knowledge of causes was measured using five‐item questions, the signs using 11 items, risk factors using 12 items, and prevention using 5 items. Each of these items carried one point when answered correctly and zero points when wrongly answered. Final scores were categorized into good knowledge (≥50% of the scores) and poor if otherwise.^13^ Attitude on cervical cancer screening was measured by asking the participant 10 questions concerning thoughts and feelings toward cervical cancer screening.^32^ The mean score was used to categorize the respondents into positive vs negative attitudes.^33^

The independent variables included social demographic characteristics and information on HIV. Social‐demographic variables included age in years, date of birth, number of children, the highest level of education (no education, primary, secondary, and higher education), marital status (single/ never married, married/cohabiting, divorced/separated/widowed, specify if others), occupation (no occupation, employed, housewife, peasant/farmer), an average woman's monthly income, and average household's monthly income (in Tanzanian Shillings). Information on HIV included the date of HIV diagnosis, the number of years since HIV diagnosis, WHO clinical stages of HIV (stage 1, stage 2, stage 3, stage 4, and not known), and the most recent CD4 count in cell/mm^3^ and if she is current on treatment.

### 2.5 Data analysis

Data cleaning and analysis were performed using SPSS version 20.0. Frequencies and percentages were used to summarize categorical variables and means/medians and standard deviations/interquartile range for numeric variables. The findings were summarized into tables, graphs, and narrations.

## 3 RESULTS

### 3.1 Participant background characteristics

A total of 303 women living with HIV met the inclusion criteria and were invited to participate in the study. Only 297 of those invited consented to participate in making a response rate of 98%. The median age of 297 women living with HIV who participated in this study was 44 (IQR 36‐49.5) years. About half (49.2%) of the women were 45 years of age or above. More than half (58.2%) were widowed/divorced or separated, and 60.6% were self‐employed. About 89.2% of the women reported having no health insurance, and only 5.1% reported a history of cervical cancer in their families (Table 1).

**TABLE 1:** Participant background characteristics (N = 297)

Variables	Frequency	Percentage
Age (years)		
<35	68	21.2
35‐44	88	29.6
≥45	146	49.2
Median (IQR)	44.0	(36.0, 49.5)^a^
Marital status		
Single	38	12.8
Married/cohabiting	86	29
Widowed/divorced/separated	173	58.2
Highest education level		
No education	12	4.0
Primary education	217	73.1
Secondary education and above	68	22.9
Occupation		
Employed	200	67.3
Peasant	72	24.2
Unemployed	25	8.4
Have health insurance		
Yes	32	10.8
No	265	89.2
Has a family history of cervical cancer		
Yes	15	5.1
No	282	94.9
Own any information technology device		
Yes	280	94.3
No	17	5.7

^a^ Interquartile range.

### 3.2 Cervical cancer screening practices

Half of all women living with HIV in this study reported having ever screened for cervical cancer. Of these, 64% were screened within the past 12 months, and 93.3% had their screening post‐HIV diagnosis. Advice from the healthcare providers was the commonly reported reason (88.6%) for cervical cancer screening in this population. Among the reasons for not being screened included having no symptoms (53.4%), unaware of where to be screened (25%), delay in obtaining service (14.5%), and fear of pain (11.5%). Less than half (48.2%) of those who ever screened reported the screening interval every once a year as per the national cervical cancer screening guidelines (Table 2).

**TABLE 2:** Cervical cancer screening practices (N = 297)

Variables	Frequency	Percentage
Ever been screened for cervical cancer		
Yes	149	50.2
No	148	49.8
Had cervical screening in the last 12 mo (n = 149)^a^		
Yes	96	64.4
No	53	35.5
Reasons for screening (n = 149)^a^ ^,^ ^b^		
HIV status	34	22.8
Advice from health care providers	132	88.6
Support from husband/sexual partner	1	0.7
Age	2	1.3
Screening campaigns	28	18.8
Reasons for not screening (n = 148)^a^ ^,^ ^b^		
Delay in obtaining service	22	14.9
No symptoms	79	53.4
Fear of results	13	8.8
Fear of pain	17	11.5
Do not know the place	37	25.0
Expensive	12	8.1
Others^c^	33	22.3
Frequency of cervical cancer screening after HIV diagnosis (n = 149)^a^		
Every 6 mo	9	6.5
Every once year	67	48.2
Less than once a year	31	22.3
I do not know	32	23.0

^a^ Variables with missing values.

^b^ Percent exceeds 100 because of multiple responses.

^c^ Being busy and not aware of it.

### 3.3 Participants knowledge of cervical cancer screening

The vast majority (89.6%) of women living with HIV in this study had ever heard of cervical cancer screening. Only a fifth (20.5%) of the participants were aware of how women living with HIV should start cervical cancer screening. About three quarters (72.1%) of the women had inadequate knowledge of cervical cancer signs and symptoms. Moreover, almost half (52.5%) had adequate knowledge of preventing cervical cancer (Table 3).

**TABLE 3:** Knowledge of cervical cancer screening (N = 297)

Variables	Frequency	Percentage
Ever heard about cervical cancer screening		
Yes	266	89.6
No	31	10.4
Awareness of the presence of a national cervical cancer screening program in Tanzania		
Yes	171	57.6
No	126	42.4
Awareness on the age at which women in the general population are supposed to start cervical cancer screening (n = 171)^a^		
Yes	16	9.4
No	155	90.6
Awareness on the timing at which women living with HIV should start cervical cancer screening		
Yes	61	20.5
No	236	79.5
Knowledge of signs and symptoms of cervical cancer		
Adequate	83	27.9
Inadequate	214	72.1
Knowledge of prevention of cervical cancer		
Adequate	156	52.5
Inadequate	141	47.5
Knowledge of risk factors of cervical cancer		
Adequate	114	38.4
Inadequate	183	61.6

^a^ Variables containing missing values.

**FIGURE 1:**
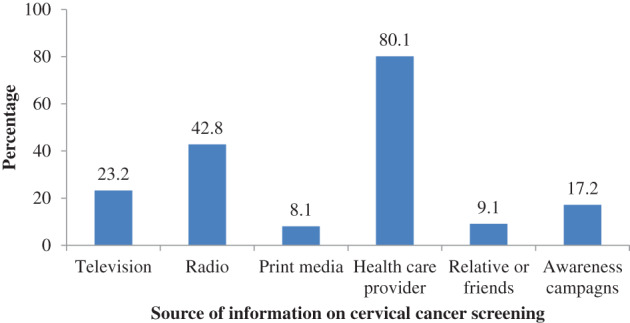
Participant's source of information on cervical cancer screening (N = 297). Percentage exceed 100 because of multiple responses

Of the 297 women living with HIV in this study, 80.1% received information on cervical cancer screening from the health care providers and only 8.1% from print media (8.1%) (Figure 1).

### 3.4 Attitude toward cervical cancer screening

The majority (88.9%) of the women living with HIV in this study saw the need for cervical cancer screening, and over three‐quarters (78.5%) were willing to be screened without having signs and symptoms. About two‐thirds (66.7%) of the women had a positive attitude toward cervical cancer screening, and 71.4% were comfortable to be screened by any health care provider regardless of their gender (Table 4).

**TABLE 4:** Attitude on cervical cancer screening (N = 297)

Variables	Frequency	Percentage
Seeing the need for cervical cancer screening		
Yes	264	88.9
No	33	11.1
The willingness of being screened		
Yes	245	82.5
No	52	17.5
Willingness to be screened for cervical cancer without having any sign or symptom of cervical cancer		
Yes	233	78.5
No	64	21.5
Fearing cervical cancer screening procedure		
Yes	45	15.2
No	252	84.8
Feeling shy to expose private parts during the procedure to young or male service provider		
Yes	60	20.2
No	237	79.8
Fear pain/discomfort during the cervical cancer screening procedure		
Yes	84	28.3
No	213	71.7
Fear of bleeding during and after cervical cancer screening procedure		
Yes	69	23.2
No	228	76.8
Fear of being diagnosed with cervical cancer after undergoing screening		
Yes	63	21.2
No	234	78.8
Think cervical cancer screening is expensive		
Yes	49	16.5
No	248	83.5
Preferred gender of provider for cervical cancer screening		
Male/female health care provider	85	28.6
Comfortable with any of them	212	71.4
Attitude toward cervical cancer screening		
Positive	198	66.7
Negative	99	33.3

## 4 DISCUSSION

We aimed to assess the knowledge, attitude, and practices on cervical cancer screening among women living with HIV in the Kilimanjaro region. This study shows that the self‐reported prevalence of cervical cancer screening among women living with HIV was 50.2%. The majority (89%) of the women had ever heard about cervical cancer screening. Still, less than half of them had adequate knowledge of cervical cancer risk factors (38.4%), sign and symptoms (27.9%), and prevention (52.5%). Moreover, only 20.5% were aware of the right time to start cervical cancer screening after HIV diagnosis. On the other hand, more than 60 % (66.7%) of all women had positive attitudes toward cervical cancer screening.

The self‐reported prevalence of cervical cancer screening among women living with HIV in this study is significantly higher than 9% among women living with HIV in Dar Es Salaam^26^ and other studies in SSA.^19^, ^31^, ^34^, ^35^, ^36^ A higher prevalence in this study might be explained by recent efforts to increase screening uptake and the integration of cervical cancer screening programs and care and treatment (CTC) services in 2017.^25^ This study's estimate is lower than that of those from high‐income countries.^37^, ^38^, ^39^ This difference can be due to delayed integration of cervical cancer screening programs with CTC services in Tanzania compared to high resourced countries.^31^

Advice from health care providers was the main reason for cervical cancer screening among women in this study, as reported in other studies.^40^, ^41^ On the other hand, about half (53.4%) of the women who have never screened reported having no symptoms and not knowing the place to go for screening, similar to findings in Ethiopia^42^ and Uganda,^19^ respectively. This implies that there is low coverage of health education programs, particularly on cervical cancer, in this population. The low cervical cancer screening among women attending CTC reflects missed opportunities. This shows a need to strengthen and encourage health care workers to create awareness and counselling services for women attending CTC.

Like Western Kenya,^3^ most participants in this study ever heard about cervical cancer screening. These findings are higher than those from other studies,^36^, ^42^ where nearly a third of the women ever heard of cervical cancer screening. This study's heightened awareness is probably due to mass awareness creation programs through media and the integration of cervical cancer screening programs with CTC services^25^ similar to Kenya.^3^ This shows a need for different countries to strengthen awareness and screening campaigns that may increase the uptake of cervical cancer screening interventions among women attending CTC and across the general population.

In this study, a fifth (20.5%) of participants were aware that cervical cancer screening should be initiated soon after HIV diagnosis, as reported in Ethiopia.^42^ Furthermore, most women in this study had inadequate knowledge of the risk factors, signs and symptoms, and cervical cancer prevention. Much lower proportions of inadequate knowledge were reported in Ethiopia^22^, ^42^ and the USA.^43^ This could have been among the reasons for the high screening uptake. The lower screening uptake in Ethiopia despite high proportions of adequate knowledge may also be due to inadequate health care provider cervical counselling on cancer screening.

Health care workers and mass media (radio and television) were the primary sources of information concerning cervical cancer screening, similar to Dar es salaam.^26^ However, this was so to only a fifth (20%) in Nigeria.^33^ A higher percentage of participants who received information from health care workers in Tanzania can also be due to the integration of cervical cancer screening programs and CTC service delivery. In Nigeria, cervical cancer information has not been incorporated into the HIV test and counselling services. Other sources of information in Nigeria included family members, relatives, and friends. This calls for strengthening the health care workers in CTCs and enhancing mass media education campaigns across the country to increase awareness and uptake of cervical cancer screening.

About two‐thirds of women living with HIV in this study had a positive attitude toward cervical cancer screening, which is significantly higher than 20.8% reported in Dar Es Salaam.^26^ The difference may be because Koneru et al^26^ conducted the study before integrating cervical cancer screening with HIV services at CTC in 2017.^25^ Furthermore, this study's estimate is higher than 43.5% reported in Nigeria but less than 87% in Ethiopia.^33^, ^42^ The noninclusion of cervical cancer information can explain Nigeria's poor attitude following the post‐HIV test counselling. The high proportion of positive attitudes reported in Ethiopia may be due to the integration of screening services in HIV management. Efforts should be made to eliminate potentially negative attitudes toward cervical cancer screening.

### 4.1 Strength and limitations

This study provides essential information about knowledge, attitudes, and cervical cancer screening practices among WLHIV after integrating cervical cancer screening with care and treatment services in Tanzania. This study has some limitations that might have affected our observed findings. Firstly, the study was hospital‐based and involved only women attending selected CTC in the Kilimanjaro region; hence, it may not represent the whole Tanzanian population. Self‐reported knowledge, attitude, and cervical cancer screening practices by study participants may be subject to reporting bias or self‐desirability bias.

## 5 CONCLUSION AND RECOMMENDATIONS

This study found that women living with HIV in this study had inadequate knowledge and favorable attitudes toward cervical cancer screening. Half of all women attending CTC in the Kilimanjaro region have ever been screened for cervical cancer, which is slightly higher than previous studies before integrating cervical cancer screening with CTC services in Tanzania and other countries SSA. Strengthening healthcare workers' capacity to promote cervical cancer awareness and adherence to screening recommendations in this population is essential to increase uptake. Furthermore, through the Ministry of Health, the government should enhance educational campaigns through different mass media such as radios, create more awareness, address potentially negative attitudes toward screening, and promote cervical cancer screening uptake among WLHIV.

## CONFLICT OF INTEREST

The authors have stated explicitly that there are no conflicts of interest in connection with this article.

## AUTHOR CONTRIBUTIONS

All authors had full access to the data in the study and take responsibility for the integrity of the data and the accuracy of the data analysis. *Conceptualization*, M.M., F.K., H.K., I.M., C.A.; *Data Curation*, M.M., F.K., H.K.; *Formal Analysis*, M.M., F.K., H.K., I.M.; *Investigation*, M.M., F.K., H.K., I.M.; *Methodology*, M.M., F.K., H.K., I.M., C.A.; *Project Administration*, M.M., F.K., H.K., I.M., C.A.; *Validation*, M.M., F.K., H.K., I.M., C.A.; *Visualization*, M.M., F.K., H.K., I.M.; *Supervision*, I.M., C.A.; *Writing‐Original Draft*, M.M., F.K., H.K., I.M.; *Writing‐Review & Editing*, M.M., F.K., H.K., I.M., C.A.

## ETHICAL STATEMENT

The study was approved by the Kilimanjaro Christian Medical University College Research and Ethics Review Committee (KCMU‐CRERC) and received an approval number UG 090/2020. Oral informed consent was obtained from participants before participation in the study. Participation in this study was voluntary and did not affect the patient's routine CTC cervices. Participants were allowed to refuse to answer any questions and terminate the interview when they desired. Confidentiality of information was ensured by using unique identifiers.

## Data Availability

The data that support the findings of this study are available from the corresponding author upon reasonable request.
